# Interplay between host and pathogen: immune defense and beyond

**DOI:** 10.1038/s12276-019-0281-8

**Published:** 2019-12-11

**Authors:** Eun-Kyeong Jo

**Affiliations:** 1 Department of Microbiology, Chungnam National University School of Medicine, Daejeon, 35015 Korea; 2 Department of Infection Control Convergence Research Center, Chungnam National University School of Medicine, Daejeon, 35015 Korea

Host–pathogen interaction is considered a highly dynamic process between diverse microbial pathogens and hosts in all stages of pathogenic infection, from invasion to dissemination. Upon pathogenic infection, innate immune systems respond to pathogen-associated molecular patterns and activate immediate host inflammatory and antimicrobial responses^1,2^. Innate immune cells trigger sophisticated intracellular signaling pathways via innate immune receptors, including membrane-bound or cytosolic receptors^1–3^. Host innate immune activation results in the production of multiple effector molecules, including cytokines and chemokines as well as antimicrobial proteins, to combat invading pathogens and parasites^4^. In addition, innate immune cells, such as macrophages and dendritic cells, are the principal antigen-presenting cells that activate T cells, which are involved in more specific and delicate immune responses^5,6^. In particular, Th1 cell-mediated interferon (IFN)-γ can efficiently promote cell-autonomous host defenses against intracellular parasitic and mycobacterial infections through various effectors, including IFN-inducible GTPases, inducible nitric oxide synthase, and autophagy proteins^7,8^.

Here, a special issue of articles presents recent findings on how host immune and pathological responses are generated by intracellular signaling machinery, which can be activated at various stages of host–pathogen interaction, and further describes how pathogens subvert host protective responses to establish an infection in the harsh environment inside host cells. In addition, this special issue includes invited reviews that discuss current knowledge of host defensive components and pathways, i.e., immune receptors, cytokines, signaling molecules, autophagy, and the microbiota. We further describe recent innovative trials based on studies of the host–pathogen interface involving the therapeutic utilization of bacteria in the context of anticancer treatments. A comprehensive understanding of host–pathogen interaction will provide new insights into the identification of novel targets for both host effectors and microbial factors and will lead to new therapeutic treatments for infections and other human diseases.

When encountering the host defense system, numerous intracellular pathogens employ a variety of evolved strategies to escape, modulate, and hijack host immunity during infection^9^. Lee and coworkers provide a comprehensive summary of viral strategies that evade host cytosolic sensing to facilitate intracellular infection and replication of viruses. Although innate immune responses are essential for combating viral infections, they should be tightly regulated to prevent harmful host responses. Lee and coworkers also review recent findings on the positive and negative regulatory mechanisms for intracellular sensors in-host cells. Recent studies have uncovered key signaling molecules that participate in the recognition of cytosolic pathogen-associated molecular patterns (PAMPs) and the activation of innate immune responses against viral infection^10,11^. Ahn and Barber highlight the immune regulatory functions of stimulators of interferon genes and cyclic dinucleotides that can recognize microbial DNA from various pathogens, including bacteria, viruses, and parasites, as well as cytosolic DNA of self-origin from host cells. Investigations on the abundance of in-host effector mechanisms and pathogenic strategies will provide an ideal opportunity to develop innovative tools that can be utilized to modulate microbial pathogenesis.

Autophagy, a cell-autonomous catabolic pathway involving lysosomal degradation of cargos, is becoming recognized as an innate effector mechanism to enhance pathogen control and resolution of tissue pathology associated with infection^12–14^. Jo and coworkers summarize the function of autophagy and xenophagy in mycobacterial infection and how to manipulate autophagy pathways to promote host-targeted therapeutics against mycobacterial infection. A more comprehensive understanding of the elaborate mechanisms by which host autophagy pathways overcome bacterial pathogenesis strategies may lead to more efficient antimicrobial defense strategies against numerous intracellular pathogens that can reside and replicate within the host cell cytoplasm^14–16^.

When considering the role of type I and II interferons (IFNs) in the activation of antiviral and Th1 immune responses, respectively, it is generally accepted that IFN actions are typically required for protective immune responses against viruses and intracellular bacteria. Yamamoto and Sasai discuss recent progress on the cell-intrinsic defense mechanisms by which IFN-inducible GTPase-dependent host defenses exhibit antiparasitic and antibacterial responses. IFN-γ-inducible GTPases, including guanylate binding proteins (GBPs) and immunity-related GTPases (IRGs), are essential in various aspects of immune protective functions, including the release of bacterial components, production of reactive nitrogen species, inflammasome activation, and autophagy^7,8,17,18^. Recent studies have also revealed the function of cytokines, including type I IFNs, IL-15, and IL-18, in the bystander activation of CD8^ +^ T cells, which are responsible for tissue injury in viral infections^19,20^. Shin and Kim will present a new concept of bystander response, which leads to pathological or protective outcomes, depending on the context. An understanding of the IFN-inducible responses in various aspects of host–pathogen interactions will be essential for the development of effective therapeutics that also minimize tissue damage.

Accumulating evidence supports the importance of the gut microbiota in human health and diseases. Takeda and Maeda summarize the recent findings of molecular details in-host-microbe interactions in rheumatoid arthritis and how alterations at the interface between the host and microbes result in excessive inflammation and infection. In addition, this review will introduce multiple metabolic and immunological mechanisms by which the gut and oral microbiota induce the development of arthritis^21^. To date, the majority of studies on host–pathogen interactions have focused on comprehensive elucidation of the innate and adaptive immune responses during infection. Recently, several efforts have been made to manipulate genetically modified bacteria for utilization as a new form of therapeutic agent for the treatment of cancer^22^. Min and coworkers review the current trials, perspectives, and limitations of therapeutic approaches using live bacteria with tumor-targeting ability and discuss recent advances in our understanding of bacterium-tumor cell interactions.

In revealing a vast array of strategies adopted by host cells and pathogens during various stages of infection, paradigms are shifting. Accumulating evidence indicates a more diverse impact of pathogenic manipulations of host cells than previously known. This special issue highlights recent advances in studies that extend traditional concepts of host–pathogen interactions toward new insights on innate and adaptive immune signaling, molecular pathogenesis, host-directed therapy, and manipulation of bacteria for anticancer therapy. We believe that the cutting-edge reviews presented here will provide novel insights into the growing area of host–pathogen interface signaling, thereby expanding basic knowledge for use in clinical applications. Studying the basic and applied fields relating to this topic will promote our knowledge of highly complex host–pathogen relationships and facilitate future investigations in this area for the development of improved therapies against infections.
